# Diversity of DNA Replication in the Archaea

**DOI:** 10.3390/genes8020056

**Published:** 2017-01-31

**Authors:** Darya Ausiannikava, Thorsten Allers

**Affiliations:** School of Life Sciences, University of Nottingham, Nottingham NG7 2UH, UK; thorsten.allers@nottingham.ac.uk

**Keywords:** DNA replication, replication origin, Orc1/Cdc6, archaea, *Sulfolobus*, *Haloferax*

## Abstract

DNA replication is arguably the most fundamental biological process. On account of their shared evolutionary ancestry, the replication machinery found in archaea is similar to that found in eukaryotes. DNA replication is initiated at origins and is highly conserved in eukaryotes, but our limited understanding of archaea has uncovered a wide diversity of replication initiation mechanisms. Archaeal origins are sequence-based, as in bacteria, but are bound by initiator proteins that share homology with the eukaryotic origin recognition complex subunit Orc1 and helicase loader Cdc6). Unlike bacteria, archaea may have multiple origins per chromosome and multiple Orc1/Cdc6 initiator proteins. There is no consensus on how these archaeal origins are recognised—some are bound by a single Orc1/Cdc6 protein while others require a multi- Orc1/Cdc6 complex. Many archaeal genomes consist of multiple parts—the main chromosome plus several megaplasmids—and in polyploid species these parts are present in multiple copies. This poses a challenge to the regulation of DNA replication. However, one archaeal species (*Haloferax volcanii*) can survive without replication origins; instead, it uses homologous recombination as an alternative mechanism of initiation. This diversity in DNA replication initiation is all the more remarkable for having been discovered in only three groups of archaea where in vivo studies are possible.

## 1. Introduction

The principles of DNA replication are common across all three domains of life—bacteria, archaea, and eukaryotes—but there is a fundamental split in terms of the machinery used [1]. The DNA replication proteins found in archaea are homologous to those of eukaryotes, but those encountered in bacteria are quite distinct [1,2]. Nevertheless, phylogenomic studies have shown that the archaeal replication machinery exhibits a striking degree of diversity. In some groups of archaea, components have been lost, while in others, a large number of additional copies have been acquired [2,3]. This is in contrast to eukaryotes where the composition of the replication complex remains constant across the domain [4].

**Figure 1:**
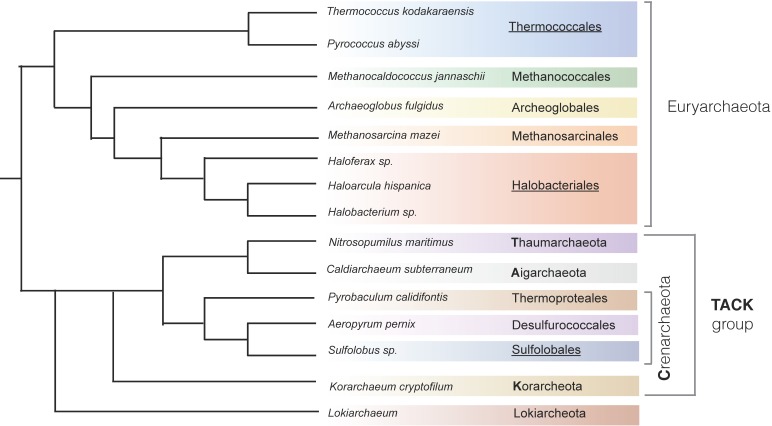
Current view of the archaeal phylogenetic tree. Based on [6,7]. The groups in which in vivo replication initiation studies have been undertaken are underlined.

Based on 16S ribosomal RNA (rRNA) sequencing, the archaeal domain was originally divided into two phyla: Crenarchaeota and Euryarchaeota [5]. However, the recent expansion in whole genome sequencing of natural isolates, combined with new statistical models, has challenged the traditional topology of the archaeal tree. It has been proposed that the TACK superphylum (comprising Thaumarchaeota, Aigarchaeota, Crenarchaeota, and Korarchaeota) gave rise to the ancestor of eukaryotes. (Figure 1). It has been suggested [3] that the diversity of replication machinery in the archaeal domain is likely to reflect the evolutionary forces that have fine-tuned their genomes in different environments.

DNA replication proceeds in three major stages: initiation, DNA synthesis, and termination. Studies of archaeal DNA replication have focused on the biochemical characterization of key enzymes involved in DNA synthesis and, despite the recognized diversity of archaeal domain, have been limited to few species. This is understandable given the interest in exploiting extremophilic enzymes in biotechnology and the difficulty of generating genetic tools for most archaeal species (see Figure 1).

DNA replication initiation is the key regulatory stage for the processes of DNA replication and the cell cycle, and the most powerful methods to study the regulation of DNA replication initiation rely on in vivo genetic analysis. However, these are available for only three groups of archaea: *Sulfolobales*, *Halobacteriales,* and *Thermococcales*. Here, we review the available knowledge on control of DNA replication initiation in archaea.

## 2. Machinery for DNA Replication Initiation

### 2.1. Replication Origins

Similar to the bacterial origins of replication, archaeal replication origins have a clearly defined structure consisting of an AT-rich DNA unwinding element (DUE) flanked by several conserved repeats termed origin recognition boxes (ORBs) that serve as binding sites for the origin recognition protein(s). The number, orientation, sequence, and spacing of ORBs vary among different genera, as reviewed in [8].

The first archaeal replication origin was experimentally identified in the *Pyrococcus* genus and it was shown to have a single origin per chromosome [9]. Since then, experimental studies and in silico predictions have identified several archaeal groups with multiple origins of replication on the same chromosome. For example, *Sulfolobus islandicus* and *Haloferax volcanii* have three replication origins per chromosome [10,11,12,13] while *Pyrobaculum calidifontis* has four, the highest number of origins per prokaryotic chromosome identified to date [14]. Interestingly, the number of origins in archaeal genomes does not correlate with genome size (Table 1). It remains an open question what advantages (if any) there are for archaeal cells in having multiple replication origins per chromosome.

### 2.2. Origin Recognition Proteins

Origins in archaea and bacteria are typically linked to the gene that encodes the replication initiator protein that recognizes the origin. In bacteria, origins are recognized by DnaA-type initiators whereas archaeal origins are recognized by Orc1/Cdc6 proteins that are homologues of the eukaryotic Orc1 origin recognition complex and Cdc6 helicase loader proteins (A confusion in naming of Orc1/Cdc6 proteins exists: in some species they are named Orc1, in others Cdc6; in essence, the same protein has homology to both to Orc1 and Cdc6). In contrast to bacteria, the proteins involved in archaeal origin recognition display a considerable degree of evolutional flexibility. *Methanococcales* and *Methanopyrales* groups have highly divergent *orc* genes that initially precluded their identification [2], while in *Sulfolobus islandicus,* the third origin of replication *oriC3* is recognized not by the Orc1/Cdc6 protein but instead by WhiP, a distant homologue of Cdt1 [18].

**Figure 2:**
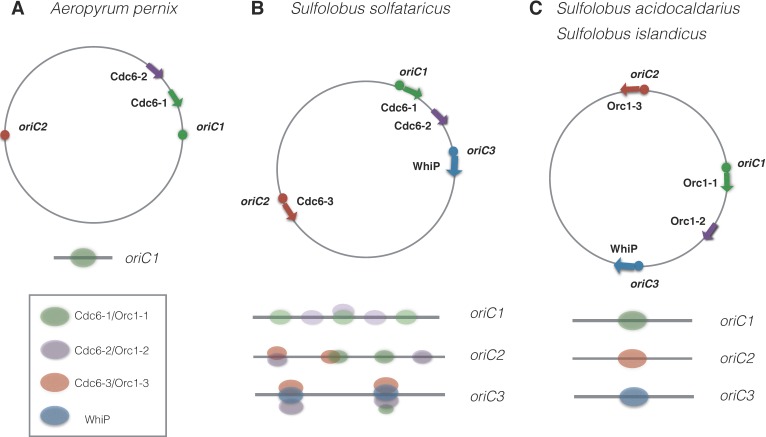
Binding of Orc1/Cdc6 proteins at origins of archaeal chromosomes. (**A**)* Aeropyrum pernix* Cdc6-1 binds to *oriC1* as a monomer; binding to the origin *oriC2* has not been investigated; (**B**) Cdc6-1, Cdc6-2, and Cdc6-3 of *Sulfolobus solfataricus* binds more than one origin each; (**C**) Replication initiation proteins of *Sulfolobus acidocaldarius* bind only one origin each. Similar to *S. acidocaldarius,* initiation proteins in *Sulfolobus islandicus* bind only one origin each.

Eukaryotic Orc proteins recognize origins as a preassembled hexameric complex, while bacterial DnaA monomers bind cooperatively to the origin of replication [1]. Most archaea encode at least two Orc1/Cdc6 homologs in their genomes, but the ability of archaeal Orc1/Cdc6 to form homo- or heteromeric complexes for origin recognition in vivo is still unclear and appears to be species-specific. The crystal structure of two Orc1/Cdc6 proteins, Cdc6-1 and Cdc6-3, bound to *Sulfolobus solfataricus* origin *oriC2* was shown to form a heterodimer [24] (Figure 2B). By contrast, the crystal structure of *Aeropyrum pernix* Cdc6-1 bound to the origin *oriC1* indicates binding as a monomer (Figure 2A) [25], while at high concentration Cdc6-1 was shown to form dimers in vitro [25,26]. The second *A. pernix* Cdc6 protein, Cdc6-2, did not bind the origin *oriC1*. Interestingly, none of the two genes for *A. pernix* Cdc6 proteins is located next to the predicted origins [19].

This notable level of diversity exists even among closely related *Sulfolobus* species. *S. solfataricus* has three replication origins (*oriC1*, *oriC2*, *oriC3*) and three Orc1/Cdc6 proteins (Cdc6-1, Cdc6-3, Cdc6-3). Deoxyribonuclease I (DNaseI) footprinting has shown that both Cdc6-1 and Cdc6-2 recognize three sites in *oriC1*, while *oriC2* and *oriC3* are recognized by all three Orc proteins, albeit with different affinities [13,19] (Figure 2B). The solved crystal structure of the Cdc6-1 and Cdc6-3 heterodimer bound to the *oriC2* origin indicates that direct contacts between Cdc6-1 and Cdc6-3 are weak, but they influence one another’s DNA binding affinities [24]. It is unclear whether Cdc6-1 and Cdc6-3 recognize the same origin independently or form preassembled complexes. Surprisingly, *oriC1* and *oriC2* origins in two related species, *Sulfolobus islandicus* and* Sulfolobus acidocaldarius,* are only bound by single Orc1-1 and Orc1-3 proteins, respectively (Figure 2C) [18,27]. However, differences in origin binding between the closely-related *Sulfolobus* species may be smaller than these studies imply and could be due to differing experimental techniques.

### 2.3. Origin Binding and DNA Unwinding

Two crystal structures are available for Orc1/Cdc6 bound to DNA: the Cdc6-1 monomer from *A. pernix* bound to *oriC1* [24] and Cdc6-1/Cdc6-3 heterodimer from *S. solfataricus* bound to *oriC2* [24]*.* Both structures indicate two general features. Firstly, limited sequence-specific interactions exist between Orc1/Cdc6 and origin DNA (four bases are contacted specifically by *A. pernix* Cdc6-1 and five bases in the case of the *S. solfataricus* Cdc6-1/Cdc6-3 heterodimer*).* Secondly, Orc1/Cdc6 proteins have bipartite DNA-interaction surfaces: the first one uses a conventional DNA-binding winged-helix domain, while the second involves the AAA+ ATPase domain. This is in contrast to DnaA, where interactions are highly sequence-specific and the ATPase domain is not involved in DNA binding.

Another aspect of Orc1/Cdc6 that differs from DnaA is the formation of higher-order complexes and their effect on DNA unwinding. DnaA binds cooperatively to multiple sites in bacterial origins and there are two reports showing cooperative binding of archaeal Orc1/Cdc6: *Methanothermobacter thermoautotrophicus* Cdc6 [28] and *A. pernix* Cdc6-1 [26]. However, DNA footprinting assays of *Sulfolobus* Orc1/Cdc6 proteins do not support the assembly of a higher-order complex on origin sites [13]. Orc1/Cdc6 have been reported to alter DNA topology in vitro [29,30] and there is one report showing origin unwinding in vitro [29]. In contrast to unwinding by bacterial DnaA, Orc1/Cdc6 were found to act in an ATP-independent manner and did not act at the duplex unwinding element. There is not yet a clear consensus on how origin DNA is unwound by Orc1/Cdc6 proteins [31].

### 2.4. Multiple Origins on the Chromosome

When multiple origins are found on archaeal chromosomes, have they arisen by duplication or do they have an independent evolutionary history? By comparing two distantly related crenarchaeal species, *Sulfolobus* and *Aeropyrum*, Robinson and Bell demonstrated that multiple origins in both species are likely to have arisen by horizontal gene transfer [19]. The authors proposed that this occurred by integration of extrachromosomal genetic elements into the chromosome, and not by the duplication of existing origins. Similar conclusions have been drawn for the multiple replication origins of haloarchaeal species, which show poor sequence similarities with each other [32,33].

The idea of replicons evolving independently of each other is consistent with in vivo studies from *Sulfolobus islandicus* and *Haloarcula hispanica*. The deletion of a single *orc1*/*cdc6* gene prevents the origin firing only from the adjacent replication origin but does not affect any other origin. Thus, only an initiation factor genetically linked to the origin is required and sufficient for the replication from that origin; the initiation of the replicons on the same chromosome is independent of each other [16,18]. The fact that *S. solfataricus* origins are bound by several Cdc6 proteins (Figure 2B) points to greater integration among the replicons in this species than in *S. islandicus* (Figure 2C). The exact combination of Orc1/Cdc6 proteins that are necessary and sufficient for origin firing in *S. solfataricus* is unknown.

### 2.5. Diversity of Functions of Orc1/Cdc6 Proteins in Archaea

The number of *orc1*/*cdc6* genes present in archaeal genomes is often greater than the number of origins. The extreme situation can be found in the *Halobacteriales* group, where the genome may contain as many as nine *orc1*/*cdc6* genes on the main chromosome but only three origins, as is the case in *Haloferax volcanii* [10,12,34]. Similar to bacterial DnaA and the eukaryotic ORC complex, archaeal Orc1/Cdc6 proteins are likely to have extended their functions beyond replication initiation. Moreover, some Orc1/Cdc6 proteins may have lost functions connected with replication initiation and have acquired new roles.

A phylogenomic analysis of 140 archaeal genomes found that in each genome, only one or two Orc1/Cdc6 homologs (named core copies) are slow-evolving, while any additional copies (shell copies) are highly divergent [3]. Shell copies of Orc1/Cdc6 might contribute to replication under special circumstances. Thus, when the three main chromosomal origins of *Haloferax mediterranei* are deleted, a dormant origin located next to the shell copy *cdc6H* gene becomes activated [15]. Alternatively, it has been suggested that Orc1/Cdc6 proteins might also work as factors for gene regulation. For example, chromatin immunoprecipitation (ChIP) analysis of *Pyrococcus abyssi* Cdc6 binding indicates that additional regions were bound in addition to *oriC1* [35]. Conversely, the transcription of shell *orc1*/*cdc6* genes was found to be misregulated when *Halobacteriales* were grown under acidic and alkaline conditions [36].

Intriguingly, when two core copies of *orc1/cdc6* are present in an archaeal genome, only one of these copies is located next to a predicted replication origin; the other *orc1*/*cdc6* gene is never linked to an origin [3]. The absence of a genetic linkage with origins suggests that the unlinked Orc1/Cdc6 proteins might have acquired functions distinct from replication initiation, for example in the regulation of gene repair, recombination, or replication fork restart. This idea is consistent with the experimental data from *Sulfolobus islandicus*, which has two slow-evolving *orc* copies, *cdc6-1* (adjacent to *oriC1*) and *cdc6-2* (not origin-associated). The deletion of *cdc6-1* inhibits the initiation from *oriC1*, while the deletion of *cdc6-2* does not affect replication initiation from any of the three origins on the chromosome (Figure 2C) [18]. However, in *S. solfataricus* the slowly-evolving Cdc6-2, which is not linked to origins, can bind to the *oriC1*, *oriC2,* and *oriC3* origins both in vivo and in vitro (Figure 2B). This has led to the proposal that Cdc6-2 can negatively regulate replication initiation [13,19].

### 2.6. Recruitment of a Helicase

The next step of replication initiation after origin recognition is recruitment of a helicase to unwind the DNA duplex. In bacteria, DnaC serves as a DnaB helicase loader, while in eukaryotes the binding of Cdc6 and Cdt1 to the ORC complex helps to recruit the minichromosome maintenance (MCM) helicase and to regulate replication initiation. In eukaryotes, the MCM helicase consists of a heterohexameric complex, whereas most archaeal MCM proteins are homohexamers encoded by a single *mcm* gene [37,38].

Which protein(s) function as a helicase loader in archaea? Archaea do not have a clear homologue of Cdt1 and Orc1/Cdc6 proteins that share homology with both Orc1 and Cdc6. Most archaea have several genes encoding Orc1/Cdc6, therefore, it has been suggested that one of these Orc1/Cdc6 proteins carries out the function of eukaryotic Cdc6 by acting as a helicase loader, while the other Orc1/Cdc6 proteins are responsible for origin recognition. Recent biochemical data support the idea that a single protein can have both Orc1 and Cdc6 features, and in at least two cases, MCM is recruited to origins directly by Orc1/Cdc6 [39,40]. In an in vitro recruitment assay, Cdc6 from *Pyrococcus furiosus* was able to recruit MCM in an *oriC*-dependent manner [40]. In *Sulfolobus islandicus,* the conserved C-terminal winged-helix domain of MCM interacts directly with the ATPase domain of Cdc6-1; this interaction is required for the replication initiation from *oriC1* in vivo [39].

It is likely that there are alternative mechanisms of MCM recruitment in archaea. For example, the MCM-interacting interface appears to be conserved in Cdc6-3, the replication initiator protein in *S. islandicus* that is required for recognition of *oriC2* [39]. By contrast, the third origin of *Sulfolobus islandicus*, *oriC3*, is bound by WhiP, a distant homolog of Cdt1 and not Orc1/Cdc6. It is likely that different interfaces of MCM are involved in its recruitment by WhiP, and that additional partners may play a role in this process [18].

An extreme case in MCM recruitment in archaea is encountered in the *Methanococcales* family. This family has very divergent Orc1/Cdc6s and several copies of MCM encoded in the genome. Although additional copies of *mcm* genes have mostly arisen by the integration of extrachromosomal elements, the mobile elements carrying these *mcm* genes do not appear to have been involved in extensive lateral gene transfer and, thus, may have coevolved with their hosts [41]. Although it is tempting to speculate that under special circumstances (e.g., replication stress) alternative MCM helicases might be recruited to the origins by different Orc1/Cdc6 proteins, the experimental evidence for this is lacking due to difficulties of genetic analysis in *Methanococcales*.

An ancient supergroup of unicellular eukaryotes called Excavates, which is comprised of *Trypanosoma, Giardia,* and *Euglena*, also lacks Cdc6 and Cdt1, and only one Orc-related initiator can be clearly identified by sequence homology [42]. Recently, Orc1/Cdc6-interacting proteins in *Trypanosoma brucei* were shown to act in nuclear DNA replication, and Orc1/Cdc6 was present in a high molecular complex suggesting the presence of a diverged ORC complex [43]. This suggests that a similar situation might exist in archaea, where at least some archaeal Orc1/Cdc6 proteins form complexes with yet-to-be identified proteins, thus increasing the efficiency of replication initiation in vivo.

## 3. Regulation of DNA Replication Initiation

In eukaryotes, strict regulation of replication initiation is required to ensure one round of chromosome replication per generation. To accomplish this, the cell must ensure that one initiation event occurs per generation per origin, and must prevent a second round of initiation. A regulated cell cycle ensures the temporal separation of DNA replication initiation (from multiple origins) and the onset of cell division, since they occur in wholly distinct phases. This is accomplished by the actions of cyclin-dependent kinases and associated factors.

Bacteria utilise another strategy for DNA replication control. The commitment to replication occurs at a single origin level, and not at a cellular level, and is determined by the concentration of active DnaA and the accessibility of the origin [44]. Thus, initiation of replication in bacteria is growth-dependent, rather than cell cycle-dependent.

Regulation of DNA replication initiation across the archaeal domain is unlikely to be uniform. Firstly, only the Crenarchaeal phylum has haploid species; all Euryarchaeal species that have been examined contain more than one copy of the genome per cell, with the number of copies being variable at different stages of growth. Secondly, species with multiple replication origins per chromosome will need to coordinate their firing. Thirdly, some domains such as *Halobacteriales* have large (up to 0.6 Mb) extrachromosomal megaplasmids that must also be replicated in a cell cycle. These diverse circumstances require a range of mechanisms to regulate DNA replication initiation.

### 3.1. Cell Cycle Regulation in Haploid Archaea

Limited knowledge of the archaeal cell cycle exists for the most studied archaeal group, the *Sulfolobus* genus, which is a haploid crenarchaeote [45]. Similar to eukaryotes, the *Sulfolobus* cell cycle is divided into pre-replicative G1 phase, S-phase where genome replication happens, post-replicative G2 phase, and M- and D phases when the genome segregation and cell division happen. The longest phase is G2, which takes more than half of the cell cycle. This is in contrast to eukaryotes, where the G2 phase is short.

One method of regulating replication initiation in eukaryotes is cell-cycle specific expression of *cdc6*. The Cdc6 helicase loader is synthesised in late G1 and recruits MCM helicase to the ORC complex in the S-phase. The pattern of Orc binding and expression differs from Cdc6, since the ORC complex is bound to DNA throughout the whole cell cycle. Given that archaeal Orc1/Cdc6 might play the role of both initiator and helicase loader, it would be interesting to know whether the level of its expression is regulated. Again, the expression pattern varies even among closely-related species. In *Sulfolobus solfataricus,* the abundance of three Cdc6 proteins appears to be cell-cycle specific and varies in a cyclin-like fashion. The expression of Cdc6 is increased in or just before the G1 phase, decreased in the S-phase, and is considerably reduced in the non-replicating stationary phase cells [13]. In *S. acidocaldarius,* the expression of Cdc6-1 and Cdc6-3, as well as their binding at origins, remains constant throughout the cell cycle as well as in the stationary phase [27]. A similar case was observed in *Pyrococcus abyssi* where Orc1/Cdc6 remains bound to the replication origin both in the exponential and stationary phases, while MCM is associated with the origin only in the exponential phase [35]. This suggests that there may be additional factors that regulate replication initiation for these species. For example, an additional component of the replication initiation machinery or post-translational modifications.

### 3.2. Cell Cycle Regulation in Polyploid Archaea

Polyploidy is widespread in the archaeal domain, for example, *Halobacteriales* and *Methanococales* are both highly polyploid [46,47]. Due to their high genome copy number, polyploid species do not have a strict requirement to replicate the genome only once per cell cycle or to evenly distribute the chromosome copies to daughter cells. In fact, it is unclear whether replication of the chromosome copies is synchronous in polyploid archaeal species. Differences in ploidy levels at different stages of growth suggest that cell division and DNA replication are not tightly coupled [46]. Whether DNA replication and cell growth are also uncoupled in archaea, as was recently reported in the polyploid cyanobacterium *Synechococcus* [48], is unknown.

### 3.3. Regulation of Initiation of Multiple Origins

Having more than one origin per chromosome potentially increases the complexity of regulation of replication initiation; this has been examined in only a few studies. In *Sulfolobus acidocaldarius*, a species with three replication origins per chromosome, there is a close coordination of firing of two origins (*oriC1* and *oriC3*) at the beginning of the S-phase, while the third origin, *oriC2,* is activated slightly later [27]. The mechanisms that ensure simultaneous origin firing are unknown.

In *Haloarcula hispanica,* a halophile with two origins per chromosome, the sequences located next to the origins appear to influence the activity of origin firing: *oriC1* has a G-rich inverted repeat that serves as an enhancer, while *oriC2* is negatively regulated by an ORB-rich region [16]. The stoichiometry between different origins might be important. *Haloarcula hispanica* wild-type cells fail to replicate a plasmid bearing an additional copy of the *oriC2* origin, while the cells lacking *oriC2* on the chromosome tolerate the plasmid-borne *oriC2* origin. This suggests that the Orc1/Cdc6 that binds to *oriC2* may be rate-limiting.

### 3.4. Regulation of Replication of Multiple Chromosomal Elements

The genomes of *Halobacteriales* consist of several parts, the main chromosome and several large extrachromosomal DNA species named megaplasmids or minichromosomes. The megaplasmids tend to have Orc1/Cdc6-based replication initiators of their own. Because the chromosome and most megaplasmids are present at a similar copy number, it is likely that for some megaplasmids there is coordination of their replication initiation with the main chromosome [10,46]. However, pHV1 (a megaplasmid found in *Haloferax volcanii*) was found to have a copy number different from that of the main chromosome, indicating that it has inputs from alternative regulation circuits [46].

## 4. Alternative Mechanisms of Replication Initiation

**Figure 3:**
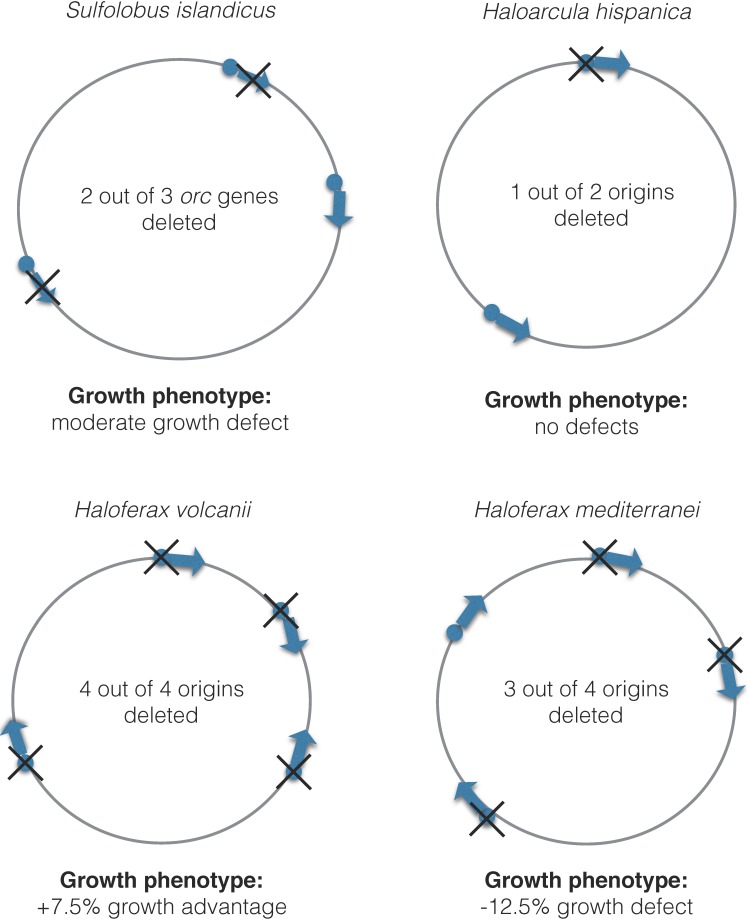
Serial deletion of *orc* genes or origins in different archaeal species. The highest number of *orc*/origin deletions possible in one strain is shown. Chromosomes are not drawn to scale.

Genetic experiments where *orc1*/*cdc6* genes and origins have been deleted suggest that replication initiation is quite flexible in archaea. The deletion of a single *orc1*/*cdc6* gene (thus inactivating the adjacent origin) in *Sulfolobus islandicus* does not affect cell growth, while the inactivation of two out of three *orc1*/*cdc6* genes leads only to a moderate growth defect. However, the deletion of all three *orc1*/*cdc6* genes is impossible [18] (Figure 3).

The consequences of deleting multiple origins or *orc1*/*cdc6* genes has also been examined in four halobacterial species: *Haloferax mediterranei, Haloferax volcanii, Haloarcula hispanica,* and* Halobacterium* NRC-1 [10,15,17,32] (Figure 3). Seven out of ten *orc1*/*cdc6* genes can be deleted simultaneously in *Halobacterium* NRC-1 [17]. In *Haloarcula hispanica*, five out of six *orc1*/*cdc6* genes located on the main chromosome and three out of four *orc1*/*cdc6* genes on the megaplasmid can be also deleted at the same time [32]. Similar to *Sulfolobus islandicus*, the deletion of one of the two origins on the main chromosome of *Haloarcula hispanica* did not lead to any growth defects under normal conditions [32]. In* Haloferax mediterranei,* it was possible to delete all three replication origins on the main chromosome, and growth of the strain lacking *oriC1*,* oriC2* and* oriC3* is 12.4% slower than the wild type [15]. However, a dormant origin, named *oriC4,* became activated in the triple origin deletion strain. As the growth defect of a triple-deleted strain suggests, this dormant origin is not able to restore growth to wild-type levels. Similar to *Sulfolobus islandicus*, the generation of a quadruple ∆*oriC* mutant was found to be impossible [15]. These studies indicate that the loss of a single *orc1*/*cdc6* gene or origin does not affect growth, while the loss of multiple *orc1*/*cdc6* genes or origins leads to slower growth, and it is impossible to delete all *orc1*/*cdc6* genes and/or origins.

However, *Haloferax volcanii* is a notable exception in this regard: the deletion of two or more origins does not result in growth defects and the deletion of all origins leads to 7.5% faster growth than wild type; however, unlike *Haloferax mediterranei*, there is no activation of dormant origins [10]. This indicates that an alternative, highly efficient mechanism for replication initiation exists in *Haloferax volcanii*. Given the common evolutionary history of *Halobacteriales*, it is likely that the core machinery for origin-independent replication exists in all species, but that *Haloferax volcanii* has lost an inhibitory component that prevents this mode of replication. Alternatively, it might have acquired an activating component that promotes origin-independent replication. Indeed, horizontal gene transfer is highly prevalent in *Halobacteriales*, as evident by a large number of gene duplications in the genome. Low species barriers exist in halophilic archaea for gene transfer and the exchange of large chromosomal fragments between *Haloferax volcanii* and *Haloferax mediterranei* has been detected in vivo [49]. Interestingly, the dormant origin that becomes activated upon deletion of three chromosomal origins in *Haloferax mediterranei* is “foreign” to its genome—its chromosomal context indicates that it was acquired during a recent lateral gene transfer event [15]. Furthermore, it is not found in *Haloferax volcanii,* which explains why it is not activated in an origin-less *Haloferax volcanii* mutant.

**Figure 4:**
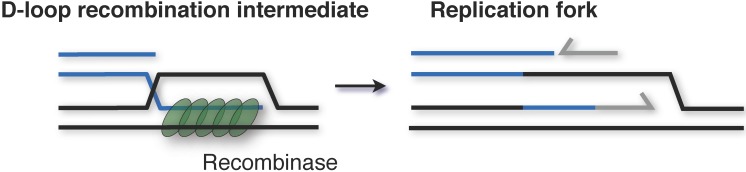
Recombination-dependent replication initiation. The invading 3′ DNA end of a displacement loop (D-loop) recombination intermediate is used as a primer for leading strand DNA synthesis. Formation of a D-loop requires a RecA-family recombinase.

Some viruses, such as bacteriophage T4, use recombination-dependent DNA replication initiation at certain life stages, where the invading 3′ DNA end of a displacement loop (D-loop) recombination intermediate is used as a primer for leading strand DNA synthesis (Figure 4). In contrast, the nuclear genomes of eukaryotes are replicated from internal origins using the replication-fork model. The case of plastids (mitochondria, chloroplasts, and kinetoplastids in *Trypanosoma*) is often overlooked. Replication is assumed to occur using the single-strand displacement model, and similar to euryarchaeal genomes, plastids contain many copies of their respective genomes. The best studied example is mitochondrial DNA replication, which begins at a site of gene transcription and proceeds unidirectionally by displacing one of the template strands as single-stranded DNA. Thus, a triple-stranded D-loop replication intermediate is formed [50].

How is recombination-dependent replication of T4 phage and single-strand displacement replication of plastids related to the origin-independent replication seen in *Haloferax volcanii*? Given that the *Haloferax volcanii* strain without origins has an absolute requirement for the recombinase RadA, it is likely that this model of replication involves D-loop intermediates that are formed by homologous recombination.

## 5. Perspectives and Open Questions

### 5.1. Tools to Control Replication Initiation in Archaeal Cells

Regulation of the cell cycle in eukaryotes is dependent on post-translational modifications of proteins. Archaea have eukaryotic-like phosphatases and kinases that may potentially phosphorylate serine, threonine, and tyrosine residues, as reviewed in [51,52]. Phosphorylation of the *Haloferax volcanii* Orc1 protein was detected by shotgun proteomic approaches [53]. The ubiquitin family of protein modification features prominently in the control of eukaryotic DNA replication [54], and ubiquitin-like small archaeal modifier proteins have been discovered in archaea [55].

Similar to eukaryotes, GTPases could be involved in mechanisms of DNA replication and repair in archaea. In several cases, genes for GTPases are located in the genomic neighbourhood of replication genes, and in *Pyrococcus abyssi,* a GTPase has been found in association with the RFC (replication factor C) clamp loader [56].

### 5.2. Spatial Organisation of Genome and Replication

In both bacteria and eukaryotes, it is well known that the three-dimensional organisation of the genome inside the cell is an important determinant in the regulation of replication. Different chromatin proteins have been described in archaea, with Alba and histone proteins being the most widespread; Alba proteins are characteristic for Crenarchaeota, while histones are found in Euryarchaeota [57]. Could it be that archaeal chromatin provides a barrier for replication fork progression, and if so, what role does it have in replication regulation? Most studies tackling this question have been focused on the Alba protein, which has been shown to exist in acetylated and non-acetylated forms; the deacetylated form represses transcription in vitro [58], and the acetylated form of Alba alleviates repression of MCM in vitro [59]. However, a direct role in DNA replication has yet to be determined.

The first attempts to correlate spatial organisation and replication have been made by Gristwood et al. [60], who used a nucleoside analogue incorporation assay to observe the sub-cellular localisation of *Sulfolobus* DNA replication. Replisomes were located at the periphery, with the three origin loci being separated in space. This suggests that replication initiation at the three origins may be regulated semi-independently.

## 6. Conclusions

The archaeal domain is the most underexplored branch of the tree of life, not only in terms of DNA replication control. Nevertheless, there are clear indications that archaea exhibit unprecedented diversity in their cellular mechanisms, while at the same time they serve as a simplified model to study many eukaryotic processes. For example, archaea with several origins per chromosome provide an excellent model for studying the coordination of replication initiation. The control of replication of polyploid archaea may give insights into DNA replication in cancer cells with multiple copies of the genome. Cell cycle studies in *Sulfolobus* can trace the development of a sophisticated cell cycle in eukaryotes. Similar arguments can be applied for unravelling connections between chromatin organisation and replication in archaea. The diversity of archaeal DNA replication resembles a melting pot of mechanisms, from which the refined system that is common to eukaryotes has emerged.

## Figures and Tables

**Table 1:** Chromosome size and number of DNA replication origins in different archaeal species.

	Chromosome Size, kb	Number of Origins per Chromosome
*Haloferax mediterranei*	2949 *	3 [15]
*Haloferax volcanii*	2848 *	3 [10]
*Haloarcula hispanica*	2995 *	2 [16]
*Halobacterium* sp.* strain NRC-1*	2014 *	2 [17]
*Nitrosopumilus maritimus*	1645	1 [14]
*Sulfolobus islandicus*	2500	3 [18]
*Sulfolobus solfataricus*	2992	3 [11,13]
*Sulfolobus acidocaldarius*	2226	3 [11]
*Aeropyrum pernix*	1670	2 [19]
*Pyrobaculum calidifontis*	2010	4 [20]
*Pyrococcus abyssi*	1770	1 [9]
*Archaeoglobus fulgidus*	2178	1 [21]
*Methanococcus jannaschii*	1660	1 ** [22]
*Methanosarcina mazei*	4096	1 ** [23]

* In cases where there are several elements of the genome, only the size of the main chromosome is indicated; ** The number of origins is based on in silico prediction by the Z-curve method and has not been experimentally validated.
